# Adverse Effects and Toxicity of Immune Checkpoint Inhibitors For Patients With Urothelial Carcinoma

**DOI:** 10.3389/fphar.2021.710943

**Published:** 2021-11-12

**Authors:** Di Wang, Kai Sun, Tianqi Wang, Dongxu Zhang, Fengze Sun, Yuanshan Cui, Hongwei Zhao, Jitao Wu

**Affiliations:** Urology Department, The Affiliated Yantai Yuhuangding Hospital of Qingdao University, Qingdao, China

**Keywords:** urothelial carcinoma (UC), immune checkpoint inhibitor (ICI), immune-related adverse events (irAEs), programmed cell death protein 1 (PD1), programmed cell death ligand protein 1 (PD-L1)

## Abstract

Urothelial carcinoma (UC) occupies a high incidence among all the genitourinary malignancies. Immune checkpoint inhibitors (ICIs), as alternative treatments of metastatic urothelial carcinoma (mUC), have been applied in the treatment of mUC after chemotherapy failure, with comparable efficacy and safety. ICIs can enhance anti-tumor T cell reactivity and promote immune control over the cancerous cells by blocking cytotoxic T-lymphocyte antigen 4 (CTLA-4) or the combination of PD-1 and PD-L1. In the treatment of urothelial carcinoma, ICIs show obvious advantage and can enhance survival rates. However, their adverse effects are gradually manifested with increasing clinical applications. Therefore, we review the adverse effects and toxicity of ICIs in patients with UC, aiming to provide sound theoretical references and therapeutic strategies for their clinical application.

## Introduction

Urothelial carcinoma (UC) is the 9th most common cancer, with a poor prognosis and chemotherapy resistance which causes a huge burden on the society and families (Richters et al., 2020; Cao et al., 2021; Sung et al., 2021). Studies have shown that patients with metastatic urothelial carcinoma (mUC) have a discouraging 5-years survival rate (Scholtes et al., 2021; von der Maase et al., 2005). Platinum-based chemotherapy is considered to be the first-line treatment for mUC, which enhances the overall response rate (ORR) by 40–60% and presents a median overall survival (OS) of 14–15 months (von der Maase et al., 2005; Logothetis et al., 1990; Loehrer et al., 1992). However, alternative treatments for patients who have failed or are resistant to chemotherapy, or who have renal function impairment, multiple comorbidities, and poor performance status are limited (Hsieh et al., 2016; Aurilio et al., 2021).

Immune checkpoint inhibitors (ICIs) can enhance the anti-tumor immune reaction of T cells by targeting cytotoxic T lymphocyte antigen-4 (CTLA-4), and programmed cell death 1 (PD-1) or its ligand (PD-L1), and have been extensively applied for various tumors, including urothelial carcinoma. Two PD-1 inhibitors (pembrolizumab and nivolumab) and three PD-L1 inhibitors (atezolizumab, avelumab, and durvalumab) have been authorized by the Food and Drug Administration (FDA) for use in mUC patients who fail to respond to chemotherapy or experience disease progression after chemotherapy (Gartrell et al., 2017; Suzman et al., 2019; Ghatalia and Plimack, 2020). In addition to single pathway blockade, combinations of anti-PD(L)1 and anti-CTLA-4 inhibitors (i.e., ipilimumab and tremelimumab) are being assessed because of potential synergistic effects of these agents. The efficacy of ICIs has been encouraging in the vast majority of patients. Moreover, ICIs combined with chemotherapy also showed better treatment outcomes for chemotherapy-eligible mUC patients compared to chemotherapy alone (Rui et al., 2019; Brown et al., 2021; Mori et al., 2021).

Meanwhile, treatment-related adverse events (trAEs) and immune-related adverse events (irAEs) caused by the use of ICIs have the subject of several studies (Friedman et al., 2016; Liu et al., 2019; Slawinski et al., 2020; Park et al., 2021). IrAEs occur when important immune system regulators are suppressed, thereby resulting in inflammatory responses in normal tissues (Apolo et al., 2017; Postow et al., 2018). Although severe complications of ICIs have been observed, irAEs are mostly low-grade and can be managed (Puzanov et al., 2017; Rapoport et al., 2017; Brahmer et al., 2018a). Therefore, this calls for the clarification of adverse events (AEs) of ICIs with the overarching goal of clinically managing mUC and further improving immunotherapeutic approaches.

In this article, we conduct an updated review of AEs in mUC patients treated with ICIs based on current clinical trial data, and discuss therapeutic strategies used to monitor and deal with such toxic reactions.

## ICIs Treatment in mUC

Significant progress has been made in the use of ICIs-based immunotherapy in cancer, either monotherapy or combination therapy. Pembrolizumab and nivolumab were the PD-1 inhibitors approved by the FDA for first-line treatment of mUC patients who were ineligible for cisplatin therapy (Balar et al., 2017a; Sharma et al., 2017). Monotherapy using pembrolizumab for mUC patients showed improvements in OS rate in the KEYNOTE-045 and KEYNOTE-052 clinical trials (Bellmunt et al., 2017; Vuky et al., 2020), and prolonged progression-free survival (PFS) in the HCRN GU14-182 trail (Galsky et al., 2020a). In addition, the KEYNOTE-361 trial showed that pembrolizumab monotherapy possessed a lower incidence of irAEs than the chemotherapy group (Alva et al., 2020). The CheckMate 032 trial showed that combination therapy had better antitumor power and controllable safety compared to monotherapy (Sharma et al., 2016; Sharma et al., 2019), while the CheckMate 275 trial concluded that nivolumab offered valuable clinical benefits (Sharma et al., 2017).

On the other hand, atezolizumab, durvalumab, and avelumab are PD-L1 inhibitors approved by the FDA for the treatment of patients with locally advanced or metastatic UC that have progressed after platinum chemotherapy or whose disease has worsened within 12 months after receiving platinum chemotherapy pre/post-operation (Balar et al., 2017b). The IMvigor211 trial demonstrated that atezolizumab is less likely to cause AEs compared to chemotherapy during treatment of mUC patients (van der Heijden et al., 2021). The IMvigor130 study reported that combining atezolizumab therapy and platinum-based chemotherapy could prolong PFS and maintain better safety compared to individual agents (Galsky et al., 2020b). Moreover, the NCT01693562 trial demonstrated that durvalumab had a significant clinical activity and controllable safety performance in patients with mUC (Massard et al., 2016; Powles et al., 2017). According to the JAVELIN study, avelumab showed safety and efficacy in post-platinum patients and cisplatin-naive patients with mUC (Patel et al., 2018). The JAVELIN Bladder 100 study marks a major breakthrough in the field of first-line treatment. For the first time in a phase 3 trial, it illustrated that immunotherapy outperforms standard care in terms of improving OS in the first-line treatment of mUC (Powles et al., 2020a). Another study named INDUCOMAIN, which we can follow up on ClinicalTrails.gov, was designed to evaluate the safety and efficacy of avelumab with gemcitabine/carboplatin versus gemcitabine/carboplatin alone in patients with mUC. This was another attempt to combine ICIs with chemotherapy for treating patients with mUC (Table 1).

**TABLE 1:** FDA approved ICIs in mUC.

Agent	Target	Approve time	Based clinical trial
Pembrolizumab	PD-1	May 18, 2017	KEYNOTE-052
Atezolizumab	PD-L1	April 17, 2017	IMvigor 210 cohort 1
Nivolumab	PD-1	February 2, 2017	CheckMate-275
Durvalumab	PD-L1	May 1, 2017	Study 1108 UC cohort
Avelumab	PD-L1	May 9, 2017	JAVELIN

CTLA-4 inhibitors mainly refer to ipilimumab and tremelimumab, which are usually conducted in clinical trials with PD1/PD-L1 inhibitors (Pardoll, 2012). In the CheckMate 032 study, two cohorts received different doses of ipilimumab plus nivolumab. The highest ORR was 38.5% for ipilimumab at 3 mg/kg and nivolumab at 1 mg/kg, and the response rate associated with ipilimumab was dose-dependent (Sharma et al., 2019). Data from the CheckMate901 study will guide whether ipilimumab in combination with nivolumab will become the standard treatment for patients with early and advanced UC. Durvalumab plus tremelimumab was another dual immune checkpoint blocking strategy, showed good clinical activity in platinum-refractory patients, particularly in patients with high PD-L1 expression (29.4 vs 15.1%). The DANUBE trial was a phase III study of durvalumab versus durvalumab plus tremelimumab versus chemotherapy in patients with advanced UC who did not receive any treatments. However, the study failed to meet its primary endpoint of improving OS when compared with single-agent durvalumab in patients with high PD-L1 expression (Powles et al., 2020b). Another clinical trial (NCT01524991) focused on durable responses and improved outcomes after treatment by gemcitabine, cisplatin, plus ipilimumab. The study enrolled 36 mUC patients, and all of them demonstrated adverse reactions in different degrees. Adverse events beyond grade 3 occurred in 81% of patients, the majority of which were hematologic (Galsky et al., 2018). However, the research did not achieve the primary endpoint. Further studies are needed to test its feasibility before widely using in similar patients. Besides combined therapy, tremelimumab monotherapy was considered to treat mUC patients regardless of prior treatments. Compared with combined therapy, CTLA-4 monotherapy did not show an encouraging result (Sharma et al., 2020).

## Mechanisms of ICIs and IrAEs

The balance of the immune system is regulated in many ways, with the negative regulation of immune inhibitor molecules being particularly important. In the initial response of T cells to antigen, CTLA-4 is upregulated on their membranes and competes with CD28 which is thought as a costimulatory factor on T cells. CD28 possesses higher affinity to B7-1 (CD80) and B7-2 (CD86) on antigen presenting cells (APCs). Unlike CD28, CTLA-4 suppresses further activity of effector T cells (Huang et al., 2017; Fritz and Lenardo, 2019). Additionally, expression of CTLA-4 on regulatory T cells (Tregs) may lead to anti-endocytosis of B7-1 and B7-2 on APCs, resulting in the absence of costimulatory factors in APCs. PD-1, which is highly expressed on the surface of most immune cells, including T cells, binds to PD-L1, which is highly expressed in antigen presenting cells (APCs) and tumor cells, thereby resulting in an inefficient T cell immune response and immune escape of tumor cells (Schreiber et al., 2011). Studies have shown that ICIs promote anti-tumor immunity by blocking the binding of PD-1 and PD-L1 (Sznol and Chen, 2013; Zhou et al., 2020). In activating T cells to exert anti-tumor effects, ICIs also allow activated T cells to cause damage to non-tumor cells, which is the main cause of irAEs.

IrAEs can occur in multiple organs, and the extent of damage varies from person to person. The severity of irAEs is assessed using the Common Term for Adverse Events Scale (CTCAE) (grade 1 indicates mild reaction, grade 2 indicates moderate reaction, grade 3 indicates severe reaction, grade 4 indicates life-threatening reaction, and grade 5 indicates death) (Brahmer et al., 2018b). Previous studies have reported that rash, pruritus, colitis, and pneumonia are the most common irAEs, which may be associated with the interaction of autoantibodies with activated immune cells during ICIs treatment (Mangan et al., 2020; Fan et al., 2021). In addition, genetic factors, host factors, and drug combinations may contribute to the development of irAEs. A previous meta-analysis concluded that ICIs combination therapy poses a high risk of irAEs compared to ICIs monotherapy (Mori et al., 2021). Among patients treated with ICIs, pneumonia is common in patients with lung cancer, while thyroiditis is frequent in patients with thyroid cancer, reflecting the association of irAEs with tumor type (Fan et al., 2021; D'Andréa et al., 2021). With the rapid increase in the use of ICIs in clinical trials, it is particularly important to identify and act against irAEs immediately.

## IrAEs in Treatment With PD1/PD-L1 Inhibitors

### IrAEs in General System

Fatigue was the most common adverse reaction in mUC patients treated with PD-1/PD-L1 inhibitors in clinical trials, ranging from 11.9 to 38.2% (Galsky et al., 2020a; Powles et al., 2020b). However, the symptom was mild in most patients, and could be managed supportively using antipyretics and antihistamines (Kumar et al., 2017). In addition, the high-grade fatigue in some patients was attributed to the digestive and endocrine system, and thus prior treatment should be directed toward the original cause. Before starting immunotherapy, laboratory tests and imaging examinations should be conducted at baseline since mUC patients may have toxic sequelae of previous treatment. The baseline results will serve as a reference for any new abnormalities that occur during immunotherapy. The most common occurrence of infusion-related reaction, with a 10.2% incidence rate, was reported in avelumab maintenance JAVELIN Bladder 100 clinical trial, but this adverse effect did not occur in any patient in the best supportive care (BSC) group (Powles et al., 2020a). Generally, infusion-related reaction often occurred during the first infusion, with the most frequent clinical manifestations being asthenia, pyrexia, influenza-like illness, dyspnea, headache, and hypotension. It is worth noting that these irAEs were mild and reversible, and could disappear after symptomatic treatment.

### IrAEs in Integumentary System

Almost all clinical trials have reported skin-related irAEs, of which the most common clinical features were rash and pruritus. In single-agent trials, rash occurred in 3–29.5% of the patients, while pruritus occurred in 3–33% of patients without dose-dependency (Plimack et al., 2017; Sharma et al., 2019). The highest frequency of both rash and pruritus was observed in the phase I/II trial named CheckMate 032, where patients received nivolumab monotherapy. There was an almost equal incidence of rash and pruritus in another group of CheckMate 032 where patients were treated using combination therapy with nivolumab and ipilimumab. However, patients who received durvalumab plus tremelimumab combination therapy had a higher incidence compared to the durvalumab monotherapy group and the chemotherapy group in the DANUBE phase III trial. In the combination therapy group, 15% of the patients reported rash and 22.9% reported pruritus, and their incidences were 7 and 10.4%, respectively, in mUC patients treated with durvalumab monotherapy. According to KEYNOTE-045 and DANUBE trials, patients in the control group who received chemotherapy showed fewer skin complications than pembrolizumab and durvalumab monotherapy patients (Bellmunt et al., 2017; Powles et al., 2020b). Moreover, cases of dry skin, dermatitis acneiform, and alopecia have been described less frequently.

Rash and pruritus often appeared at the start of ICIs therapy and may last for several months. The most common places affected by pruritus were the torso and extremities (Kamińska-Winciorek et al., 2019). Before diagnosing the skin-related irAEs, researchers should eliminate other influences, such as the basic condition of patients and side effects of other drugs. Fortunately, the majority of dermatological trAEs were mild (grade1-2), and thus treatment with ICIs can be continued. Topical emollients, topical mild-strength corticosteroids, and oral antihistamines were applied for patients who could not tolerate mild skin toxicities. When severe skin toxicities appeared, patients were treated with oral glucocorticoids and biopsy was also considered (Thompson et al., 2019). To some extent, findings from these clinical trials revealed that skin-related AEs may indicate a great prognosis.

### IrAEs in Gastrointestinal System

Diarrhea, nausea, decreased appetite, and constipation are common adverse effects of the gastrointestinal system. The onset of those symptoms occurred relatively quickly, usually before 6 weeks. During ICIs monotherapy, diarrhea occurred in 6–36.4% of patients, 5–16.4% of the patients had nausea, 4.4–13.7% of patients had decreased appetite, 2.3–23.6% of patients had constipation, and vomiting occurred in 3.5–17% of the patients (Balar et al., 2017b; Bellmunt et al., 2017; Patel et al., 2018; Galsky et al., 2020a; Powles et al., 2020a; Vuky et al., 2020). Although most of them were assessed at a grade of 1–2, there were some instances of grade 3–4 in all those symptoms. In grade 3–4 AEs observed in the CheckMate 032 trial, diarrhea ranked first with an incidence of 9.8% in a group where patients received four doses of nivolumab 1 mg/kg plus ipilimumab 3 mg/kg. Chemotherapy resulted in a higher frequency of digestive toxicity, but such cases were not more severe than toxicities associated with ICIs monotherapy. The highest incidence of diarrhea and vomiting was observed in the phase II study HCRN GU14-182, which had demonstrated kinds of electrolyte disturbance, including hyperkalemia, hypercalcemia, hypoalbuminemia, hypomagnesemia, and hyponatremia. Furthermore, three patients with gastroesophageal reflux disease were reported in the same study. Although the study report did not analyze the connection between those trAEs, diarrhea and vomiting accounted for a large part of the reason. Among all the trials, some digestive toxicities occurred less frequently, such as colitis (autoimmune colitis and enterocolitis) and an increased level of pancreatic enzymes like lipase and amylase. Early diagnosis and treatment of colitis was particularly important because severe colitis could lead to colonic perforation and peritonitis. The IMvigor 210 cohort 2 study reported that colitis occurred in three patients. Notably, colonoscopy is the gold standard for confirming colitis (Abu-Sbeih et al., 2018). When normal mucous membranes, mild erythema with mucous granules, and/or severe ulcer appeared, the diagnosis should be highly taken into account.

The treatment of gastrointestinal AEs depends on the intensity of symptoms. In instances where diarrhea was reported, researchers must exclude other diagnoses, including infections and immune-mediated toxicity such as hyperthyroidism, using laboratory examinations. Adverse reactions of the gastrointestinal system could easily lead to electrolyte disturbance, thus antidiarrheal rehydration and electrolyte replacement should be applied for mild-toxicity patients, and administration of ICIs can be continued (Thompson et al., 2020). Furthermore, treatment with glucocorticoids should be considered for worsening and persistent symptoms. If oral and intravenous glucocorticoids failed to relieve symptoms, then infliximab should be given at a dose of 5 mg/kg every 2 weeks (Cramer and Bresalier, 2017). It is worth noting that prolonged courses of glucocorticoids are preferred compared to infliximab therapy.

### IrAEs in Hepatic System

The studies reported varying clinical manifestations of liver-related AEs, including increased aspartate aminotransferase (AST), alanine aminotransferase (ALT), alkaline phosphatase, bilirubin, and cholestatic hepatitis. In the 1108 UC cohort study, autoimmune hepatitis occurred in two patients, with one of them being beyond grade 3 (Powles et al., 2017). The severity of hepatotoxicity was assessed using biochemical hepatic indicators. Among patients that received ICIs monotherapy treatment, elevated AST and ALT of all grades were reported in 0.8–22% and 0.4–18% patients, respectively (Patel et al., 2018; Galsky et al., 2020a). The HCRN GU14-182 study revealed that 22% of the patients experienced increased AST (5% of them were beyond grade 3) and 18% of patients experienced increased ALT (6% of them were beyond grade 3). Moreover, two deaths associated with hepatitis were reported in HCRN GU14-182 and DANUBE trials where patients received pembrolizumab maintenance therapy and durvalumab monotherapy, respectively (Galsky et al., 2020a; Powles et al., 2020b). Another death resulting from acute hepatic failure was also reported in the DANUBE trial.

IrAEs in hepatic system usually appeared at 4–6 weeks after commencing ICIs therapy. To reduce the incidence of liver toxicity, liver function must be checked before each infusion. Glucocorticoid is the main drug for treating hepatotoxicity. In instances where the liver function gets worse during the treatment, intensive treatment measures should be considered, such as changing oral medication to intravenous medication. Severe hepatotoxicity requires high-dose of glucocorticoids (intravenous) for 1–2 days, followed by a slow taper for the next months. Notably, stopping glucocorticoids therapy suddenly is not allowed.

### IrAEs in Endocrine System

Hypothyroidism is the most common endocrine irAEs in ICIs treatment. In patients receiving pembrolizumab therapy, the incidence of hypothyroidism ranged from 6.4 to 10.0%, while patients receiving chemotherapy therapy had an incidence of only 1.2% (Bellmunt et al., 2017; Vuky et al., 2020). This adverse effect also occurred in 7% of patients treated with atezolizumab. Patients receiving atezolizumab monotherapy presented a lower incidence about 7.7%, while the combined therapy group (nivolumab plus ipilimumab) presented an incidence about 13.0% (Sharma et al., 2019). In addition, hypothyroidism occurred in 4% of patients receiving avelumab monotherapy and in 11.6% of patients receiving avelumab maintenance therapy. There were no related AEs found in patients with durvalumab treatment. Fatigue usually manifested when hypothyroidism occurred. One patient receiving avelumab monotherapy experienced adrenal insufficiency, which manifested at grade 3.

Generally, hypothyroidism can be diagnosed by detecting serum thyrotropin (TSH) level, but its clinical manifestations are easily ignored by tumors. Patients should obtain treatment when related symptoms are observed or when the TSH level is beyond 10 mIU/L. Such patients should be subjected to a 0.5–1 μg/kg dose of l-thyroxine. In addition, up to 50% of patients with irreversible thyroid function injury need lifelong hormone replacement therapy (Haanen et al., 2018). High-dose glucocorticoid is unconventional because the curative effect is not accurate, and thus it should only be used in severe cases. With regard to patients with adrenal insufficiency, intravenous rehydration and corticosteroids should be started immediately when adrenal crisis happens (Thompson et al., 2019).

### IrAEs in Respiratory System

The clinical manifestations of irAEs in the respiratory system featured as pneumonitis cough and nasal congestion. Approximately 4% of the patients experienced pneumonitis during ICIs therapy. Although the incidence of pneumonia was relatively lower than other irAEs, it represented a large proportion of deaths associated with irAEs. Pneumonitis leading to death occurred in one patient in the KEYNOTE-045 with pembrolizumab monotherapy. In the CheckMate 032 and CheckMate 275 trials, two patients receiving nivolumab monotherapy and one patient with nivolumab plus ipilimumab combined therapy experienced pneumonitis, which eventually led to death (Bellmunt et al., 2017; Sharma et al., 2017; Sharma et al., 2019). Moreover, two patients with durvalumab therapy and one patient with durvalumab plus tremelimumab therapy died due to pneumonitis. In the avelumab monotherapy trial called JAVELIN solid tumor, pneumonitis leading to death occurred in one patient. Another adverse effect was cough, with an incidence rate ranging from 3.2 to 27% during ICIs therapy. However, cough is a symptom of pneumonia, and thus it is difficult to distinguish it.

Although the incidence of irAEs in the respiratory system was lower, pneumonitis proved to be fatal. Therefore, early diagnosis is vital for improving the prognosis. Generally, if patients presented respiratory symptoms like cough and dyspnea, it suggests lesions in the lungs, and thus chest X-ray and CT scans should be performed as routine imaging examinations. In addition, 1 mg/kg/day of oral steroid prednisone should be administered to treat patients with ICIs related grade 1–2 pneumonia. On the other hand, ICIs therapy should be immediately discontinued once the pneumonia reaches grade 3–4, and intravenous methylprednisolone at a rate of 1–2 mg/kg/day can be considered (Thompson et al., 2019). In instances where there is no improvement in the symptoms, physicians can add immunosuppressive therapy, such as infliximab, mycophenolate mofetil (MMF), or cyclophosphamide (Puzanov et al., 2017).

### IrAEs in Other Systems

There was a wide variation of manifestations in the hematological system, including anemia, thrombocytopenia, leukopenia, and neutropenia. Results showed that there was a lower incidence of patients with anemia in the ICIs therapy group compared to the chemotherapy group. In the KEYNOTE-045 study, the incidence of anemia was 3.4% in the pembrolizumab group versus 24.7% in the chemotherapy group. The same phenomenon was observed in the DANUBE study, where the incidence in the durvalumab group and the chemotherapy group were 1.7 and 41.9%, respectively, but most of them were at a low grade (Bellmunt et al., 2017; Powles et al., 2020b). The therapy focused on those toxicities was symptomatic treatment. Furthermore, glucocorticoids and alternative immunosuppression could be considered to treat refractory patients.

With regard to the urinary system, hematuria and urinary tract infections (UTI) were reported in the HCRN GU14-182 and JAVELIN Bladder 100 studies where patients received ICIs maintenance therapy. However, there were no significant differences between the ICIs group and the control group. Notably, one patient experienced sepsis leading to death after receiving eleven infusions of avelumab, which can be attributed to UTI (Powles et al., 2020a). Acute kidney injury occurred in one patient in a study involving 1,108 patients. For such patients, renal function should be checked regularly and immunotherapy should be suspended or stopped permanently according to the severity.

As for neurological adverse effects, peripheral neuropathy (including peripheral sensory neuropathy) occurred in 1.2% of patients with pembrolizumab therapy and 21.6% of patients with chemotherapy therapy in the KEYNOTE-045 study, and 2.8% of patients in the chemotherapy group were beyond grade 3. Neuromyopathy and toxic encephalopathy occurred in two patients in the KEYNOTE-012 study. In addition, one patient in the JAVELIN solid tumor study experienced Guillain Barré syndrome. To treat the mild neurotoxicity, patients should receive corticosteroid therapy, either oral or intravenous, at a dose of 0.5–1 mg/kg. Once the Guillain Barré syndrome is diagnosed, ICIs treatment must be discontinued, and plasmapheresis or immunoglobulins should be considered (Puzanov et al., 2017).

The incidence of arthralgia, myalgia, and myositis was not higher than 16, 9, and 6% in the ICIs monotherapy, and most of them were at a low grade. Paracetamol and non-steroidal anti-inflammatory drugs (NSAID) could be used to treat those symptoms. The occurrence of cardiotoxicity was rare in those trials, but it is worth noting that one 68-year-old man died of cardiovascular failure 16 days after the first nivolumab infusion (Sharma et al., 2017) (Tables 2, 3).

**TABLE 2:** Key clinical trials’ efficacy data of ICIs in patients with mUC.

Agent	Clinical trial	Phase	Line of therapy	Treatment arms	Efficacy outcomes	Median follow-up time	Publish year	Status
Pembrolizumab	KEYNOTE-012	Phase b	First line	Pembrolizumab 10 mg/kg every 2 weeks	ORR 26%	13 months	2017	Completed
					mPFS 2 months			
					mOS 13 months			
					mDoR 10 months			
	KEYNOTE-045	Phase III	Second line	Pembrolizumab 200 mg every 3 weeks	mOS 10.3 months	14.1 months	2017	Completed
					mPFS 2.1 months			
					ORR 21.1%			
					mDoR 3.5 months			
				Investigator’s choice of chemotherapy with paclitaxel, docetaxel, or vinflunine	mOS 7.4 months			
					mPFS 3.3 months			
					ORR 11.4%			
					mDoR 1.5 months			
	KEYNOTE-052 (long-term outcomes)	Phase II	First line	Pembrolizumab 200 mg every 3 weeks	ORR 28.6%	29.3 months	2020	Active, not recruiting
					mPFS 2.1 month			
					mOS 11.3 months			
					mDoR 30.1 months			
	KEYNOTE-361	Phase III	First line	Pembrolizumab plus chemotherapy	ORR 54.7%	NR	2020	Active, not recruiting
					mPFS 8.3 months			
					mOS 17.0 months			
					mDoR 8.5 months			
				Pembrolizumab	ORR 30.3%			
					mPFS 3.9 months			
					mOS 15.6 months			
					mDoR 28.2 months			
				Chemotherapy	ORR 44.9%			
					mPFS 7.1 months			
					mOS 14.3 months			
					mDoR 6.2 months			
	HCRN GU14-182	Phase II	Maintenance therapy	Pembrolizumab 200 mg every 3 weeks for up to 24 months	ORR 23.0%	12.9 months	2020	Active, not recruiting
					mPFS 5.4 months			
					mOS 22.0 months			
				Placebo	ORR 10%			
					mPFS 3.0 months			
					mOS 18.7 months			
Atezolizumab	IMvigor 210 cohort 1	Phase II	First line	Atezolizumab 1,200 mg every 3 weeks	ORR 23%	17.2 months	2017	Active, not recruiting
					mPFS 2.7 months			
					mOS 15.9 months			
					mDoR NR			
	IMvigor 210 cohort 2	Phase II	Second line	Atezolizumab 1,200 mg every 3 weeks	ORR 15%	11.7 months	2017	Active, not recruiting
					mPFS 2.1 months			
					mOS 7.9 months			
					mDoR NR			
	IMvigor 211 (long-term outcomes)	Phase III	Second line	Atezolizumab 1,200 mg every 3 weeks	24-months OS rate 23%	33.0 months	2021	Completed
				Investigator’s choice of chemotherapy with paclitaxel, docetaxel, or vinflunine	24-months OS rate 13%			
	IMvigor 130	Phase III	First line	Atezolizumab plus platinum-based chemotherapy	ORR 47%	11.8 months	2020	Active, not recruiting
					mPFS 8.2 months			
					mDoR 8.5 months			
				Atezolizumab 1,200 mg monotherapy	ORR 23%			
					mOS 15.7 months			
					mDoR NR			
				Placebo plus platinum-based chemotherapy	ORR 44%			
					mPFS 6.3 months			
					mOS 13.1 months			
					mDoR 7.6 months			
Nivolumab	CheckMate 032 (expansion cohort results)	Phase I/II	Second line	Nivolumab 3 mg/kg monotherapy every 2 weeks (N3)	ORR 25.6%	24.5 months	2019	Recruiting
					mPFS 2.8 months			
					mOS 9.9 months			
				Nivolumab 3 mg/kg plus ipilimumab 1 mg/kg every 3 weeks for four doses followed by nivolumab monotherapy 3 mg/kg every 2 weeks (N3I1)	ORR 26.9%			
					mPFS 2.6 months			
					mOS 7.4 months			
				Nivolumab 1 mg/kg plus ipilimumab 3 mg/kg every 3 weeks for four doses followed by nivolumab monotherapy 3 mg/kg every 2 weeks (N1I3)	ORR 38.0%			
					mPFS 4.9 months			
					mOS 15.3 months			
	CheckMate 275	Phase II	Second line	Nivolumab 3 mg/kg every 2 weeks	ORR 19.6%	7.0 months	2017	Active, not recruiting
					mPFS 2.0 months			
					mOS 8.7 months			
					mDoR NR			
Durvalumab	Study 1108 UC cohort	Phase I/II	Second line	Durvalumab 10 mg/kg every 2 weeks for up to 12 months	ORR 17.8%	5.8 months	2017	Completed
					mPFS 1.5 months			
					mOS 18.2 months			
					mDoR NR			
	DANUBE	Phase III	First line	Durvalumab 1,500 mg every 4 weeks	ORR 25.7%	41.2 months	2020	Active, not recruiting
					mPFS 2.3 months			
					mOS 13.2 months			
					mDoR 9.3 months			
				Durvalumab 1,500 mg plus tremelimumab 75 mg every 4 weeks for four doses, followed by durvalumab maintenance (1,500 mg) every 4 weeks	ORR 36.3%			
					mPFS 3.7 months			
					mOS 15.1 months			
					mDoR 11.1 months			
				Chemotherapy for up to six cycles	ORR 49.1%			
					mPFS 6.7 months			
					mOS 12.1 months			
					mDoR 5.7 months			
	NCT02812420	Phase Ⅰ	Second line	Durvalumab (1,500 mg kg^−1^) and tremelimumab (75 mg kg^−1^) every 4 weeks	Incidence of AEs	19.2 months	2020	Active, not recruiting
Avelumab	JAVELIN solid tumor	Phase Ⅰ	Second line	Avelumab 10 mg/kg every 2 weeks	ORR 17%	9.9 months	2017	Completed
					mPFS 1.5 months			
					mOS 6.5 months			
					mDoR NR			
	JAVELIN Bladder 100	Phase III	Maintenance therapy	Avelumab maintenance therapy 10 mg/kg every 2 weeks	ORR 9.7%	≥19 months	2020	Active, not recruiting
					mPFS 3.7 months			
					mOS 21.4 months			
					mDoR 24.9 weeks			
				Best supportive care alone (BSC)	ORR 1.4%			
					mPFS 2.0 months			
					mOS 14.3 months			
					mDoR 13.1 weeks			
Ipilimumab	NCT01524991	Phase II	First line	Two cycles of gemcitabine plus cisplatin (GC) followed by four cycles of GC plus ipilimumab	ORR 69%	NR	2017	Completed
					mPFS 7.9 months			
					mOS 13.9 months			
					mDoR 24.9 weeks			
Tremelimumab	NCT02527434	Phase II	Second line	Tremelimumab monotherapy	ORR 18.8%	9.3 months	2019	Active, not recruiting

Abbreviations: ORR, overall response rate; mPFS, median progression-free survival; mOS, median overall survival; mDoR, median duration of response; NR, not reached or not reported.

**TABLE 3:** Key clinical trials’ safety data of ICIs in patients with mUC.

Agent	Clinical trial	Treatment arms	Enrolled patients	AEs	Most common AEs	AEs (grade ≥3)	Most common AEs (grade ≥3)	Death related to AEs
Pembrolizumab	KEYNOTE-012	Pembrolizumab 10 mg/kg every 2 weeks	33	60.6%	Fatigue (18.2%)	15.2%	Increased ALT, myalgia, myositis, dehydration, hypercalcaemia, thrombocytopenia, rhabdomyolysis, neuromypathy, toxic encephalopathy, maculopapular rash, pruritic rash (3.0%, respectively)	NONE
					Peripheral oedema (12.1%)			
					Increased ALT (6.1%)			
					Myalgia (6.1%)			
					Myositis (6.1%)			
	KEYNOTE-045	Pembrolizumab 200 mg every 3 weeks	270	60.9%	Pruritus (19.5%)	14.8%	Fatigue (1.1%)	Pneumonitis, *n* = 1
					Fatigue (13.9%)		Diarrhea (1.1%)	
					Nausea (10.9%)		Anemia (0.8%)	
					Diarrhea (9.0%)			
		Investigator’s choice of chemotherapy with paclitaxel, docetaxel, or vinflunine	272	90.2%	Alopecia (37.6%)	49.4%	Neutropenia (13.3%)	Sepsis, *n* = 2 Septic shock, *n* = 1 Unspecified cause, *n* = 1
					Fatigue (27.8%)		Neutrophil count decreased (12.2%)	
					Anemia (24.7%)		Anemia (7.8%)	
					Nausea (24.3%)		Fatigue (4.3%)	
					Constipation (20.4%)		Constipation (3.1%)	
	KEYNOTE-052 (long-term outcomes)	Pembrolizumab 200 mg every 3 weeks	370	67.3%	Fatigue (18.1%)	NR	NR	Myositis, *n* = 1
					Pruritus (17.8%)			
					Rash (11.6%)			
					Decreased appetite (10.8%)			
					Hypothyroidism (10.0%)			
	KEYNOTE-361	Pembrolizumab plus chemotherapy	351	NR	NR	75.1%	NR	NR
		Pembrolizumab	307	NR	NR	16.9%	NR	NR
		Chemotherapy	352	NR	NR	71.6%	NR	NR
	HCRN GU14-182	Pembrolizumab 200 mg every 3 weeks for up to 24 months	55	90.9%	Fatigue (38.2%)	58.2%	Hyperglycemia (10.9%)	Hepatitis, *n* = 1
					Diarrhea (36.4%)		Anemia (9.1%)	
					Anemia (30.9%)		Hypertension (9.1%)	
					Creatinine increased (30.9%)		Fatigue (7.3%)	
					Lymphocyte count decreased (30.9%)		Urinary tract infection (7.3%)	
		Placebo or cross over to receive open-label pembrolizumab	52	92.3%	Fatigue (38.5%)	38.5%	Anemia (9.6%)	NONE
					Anemia (36.5%)		Hypertension (9.6%)	
					Creatinine increased (23.1%)			
							Lymphocyte count decreased (7.7%)	
					Constipation (23.1%)		Dehydration (3.8%)	
					Nausea (21.2%)			
					Hypertension (21.2%)			
Atezolizumab	IMvigor 210 cohort 1	Atezolizumab 1,200 mg every 3 weeks	119	66.4%	Fatigue (30.3%)	16.0%	Fatigue (3.4%)	NONE
					Diarrhea (11.8%)		Increased ALT (3.4%)	
					Pruritus (10.9%)		Increased AST (2.5%)	
					Decreased appetite (9.2%)			
					Hypothyoidism (6.7%)			
	IMvigor 210 cohort 2	Atezolizumab 1,200 mg every 3 weeks	310	69.4%	Fatigue (30%)	16.1%	Fatigue (1.6%)	NONE
					Nausea (13.5%)		Anemia (1.0%)	
					Decreased appetite (11.6%)		Hypertension (1.0%)	
					Pruritis (10.0%)			
					Pyrexia (9.0%)			
	IMvigor 211 (long-term outcomes)	Atezolizumab 1,200 mg every 3 weeks	459	70.4%	NR	22.2%	Anaemia (9.6%)	NONE
							Urinary tract infection (5.2%)	
							Fatigue (4.4%)	
							Asthenia (3.9%)	
							Neutropenia (0.7%)	
		Investigator’s choice of chemotherapy with paclitaxel, docetaxel, or vinflunine	443	89.2%	NR	44.5%	Neutropenia (11.1%)	NONE
							Anaemia (9.0%)	
							Febrile neutropenia (6.3%)	
							Fatigue (5.6%)	
							Neutrophile count decreased (5.6%)	
	IMvigor 130	Atezolizumab plus platinum-based chemotherapy	453	95.8%	NR	NR	NR	NONE
		Atezolizumab 1,200 mg monotherapy	354	59.6%	NR	NR	NR	NONE
		Placebo plus platinum-based chemotherapy	390	95.6%	NR	NR	NR	NONE
Nivolumab	CheckMate 032 (expansion cohort results)	Nivolumab 3 mg/kg monotherapy every 2 weeks (N3)	78	84.6%	Fatigue (35.9%)	28.2%	Lipase increased (6.4%)	NONE
					Pruritus (33.3%)		Amylase increased (5.1%)	
					Maculopapular rash (21.8%)		Maculopapular rash (3.8%)	
					Lipase increased (16.7%)		Fatigue (2.3%)	
					Arthralgia (15.4%)		Dyspnea (2.3%)	
		Nivolumab 3 mg/kg plus ipilimumab 1 mg/kg every 3 weeks for four doses followed by nivolumab monotherapy 3 mg/kg every 2 weeks (N3I1)	104	84.6%	Fatigue (31.7%)	31.7%	Increased ALT (5.8%)	NONE
					Pruritus (28.8%)		Lipase increased (5.8%)	
					Diarrhea (23.1%)		Diarrhea (4.8%)	
					Increased ALT (19.2%)		Increased AST (3.8%)	
					Maculopapular rash (18.3%)		Fatigue (2.9%)	
		Nivolumab 1 mg/kg plus ipilimumab 3 mg/kg every 3 weeks for four doses followed by nivolumab monotherapy 3 mg/kg every 2 weeks (N1I3)	92	80.4%	Diarrhea (32.6%)	39.1%	Diarrhea (9.8%)	NONE
							Increased ALT (6.5%)	
					Pruritus (31.5%)			
					Fatigue (26.1%)		Lipase increased (4.3%)	
					Decreased appetite (16.3%)			
							Maculopapular rash (3.3%)	
					Maculopapular rash (16.3%)			
	CheckMate 275	Nivolumab 3 mg/kg every 2 weeks	270	64.4%	Fatigue (16.7%)	17.8%	Fatigue (1.9%)	Pneumonitis, *n* = 1 Acute respiratory failure, *n* = 1 Cardiovascular failure, *n* = 1
					Pruritus (9.3%)		Diarrhea (1.9%)	
					Diarrhea (8.9%)		Asthenia (1.5%)	
					Decreased appetite (8.1%)		Rash (1.1%)	
					Hypothyoidism (7.8%)		Nausea (0.4%)	
Durvalumab	Study 1108 UC cohort	Durvalumab 10 mg/kg every 2 weeks for up to 12 months	191	60.7%	Fatigue (19.4%)	6.8%	Increased AST level (1.6%)	Autoimmune hepatitis, *n* = 1 Pneumonitis, *n* = 1
					Decreased appetite (9.4%)			
							Increased ALT level (1.0%)	
					Diarrhea (8.9%)			
					Rash (7.3%)		Increased GGT level (1.0%)	
					Nausea (6.8%)			
							Hypertension (1.0%)	
	DANUBE	Durvalumab 1,500 mg every 4 weeks	345	55.9%	Fatigue (11.9%)	14.2%	Lipase increased (2.0%)	Acute hepatic failure, *n* = 1 Hepatitis, *n* = 1
					Pruritus (10.4%)			
					Nausea (7.8%)		Anaemia (1.2%)	
					Decreased appetite (6.7%)		Amylase increased (0.9%)	
					Diarrhea (6.7%)		Decreased appetite (0.9%)	
		Durvalumab 1,500 mg plus tremelimumab 75 mg every 4 weeks for four doses, followed by durvalumab maintenance (1,500 mg) every 4 weeks	340	74.7%	Pruritus (22.9%)	27.9%	Lipase increased (4.7%)	Septic shock, *n* = 1 Pneumonitis, *n* = 1
					Diarrhea (21.1%)		Diarrhea (2.6%)	
					Rash (15%)		Amylase increased (2.4%)	
					Fatigue (14.4%)		Fatigue (1.8%)	
					Decreased appetite (7.6%)		Asthenia (1.5%)	
		Chemotherapy for up to six cycles	313	90.1%	Anaemia (41.9%)	60.4%	Neutropenia (21.1%)	Acute kidney injury, *n* = 1
					Nausea (40.9%)		Anaemia (19.8%)	
					Fatigue (27.2%)		Decreased neutrophile count (14.7%)	
					Neutropenia (26.5%)		Decreased platelet count (9.9%)	
					Decreased appetite (19.2%)		Thrombocytopenia (7.7%)	
	NCT02812420	Durvalumab (1,500 mg kg^−1^) and tremelimumab (75 mg kg^−1^) every 4 weeks	28	92.9%	Amylase increased (28.6%)	21.4%	Lipase increased (14.3%)	NONE
							Alanine aminotransferase increased (7.1%)	
					Rash (28.6%)			
					Pruritus (25%)			
							Aspartate aminotransferase increased (7.1%)	
					Alanine aminotransferase increased (21.4%)			
					Aspartate aminotransferase increased (21.4%)			
Avelumab	JAVELIN solid tumor	Avelumab 10 mg/kg every 2 weeks	249	66.7%	Fatigue (16.1%)	7.2%	Fatigue (1.6%)	Pneumonitis, *n* = 1
					Rash (14.9%)		Asthenia (0.8%)	
					Diarrhea (6.0%)		Lipase increased (0.8%)	
					Asthenia (5.2%)			
					Decreased appetite (4.4%)		Hypophosphataemia (0.8%)	
	JAVELIN Bladder 100	Avelumab maintenance therapy 10 mg/kg every 2 weeks	344	98.0%	Fatigue (17.7%)	47.4%	Urinary tract infection (4.4%)	Sepsis, *n* = 1 Ischemic stroke, *n* = 1
					Pruritus (17.2%)			
					Urinary tract infection (17.2%)		Anemia (3.8%)	
							Fatigue (1.7%)	
							Hematuria (1.7%)	
					Diarrhea (16.6%)		Back pain (1.2%)	
							Vomiting (1.2%)	
					Arthralgia (16.3%)			
					Asthenia (16.3%)			
					Constipation (16.3%)			
		Best supportive care alone (BSC)	345	77.7%	Hematuria (10.7%)	25.2%	Anemia (2.9%)	NONE
					Urinary tract infection (10.4%)		Urinary tract infection (2.6%)	
					Back pain (9.9%)		Back pain (2.3%)	
					Constipation (9.0%)		Hematuria (1.4%)	
					Fatigue (7.0%)		Asthenia (1.2%)	
Ipilimumab	NCT01524991	Two cycles of gemcitabine plus cisplatin (GC) followed by four cycles of GC plus ipilimumab	36	100%	Fatigue (91.7%)	80.6%	Neutrophil count decreased (36.1%)	NONE
					Nausea (75%)			
					Constipation (66.7%)		Anemia (10%)	
							Platelet count decreased (19.4%)	
					Anemia (66.7%)			
					Diarrhea (63.9%)		Hypokalemia (11.1%)	
							Thromboembolic event (11.1%)	
							Diarrhea (11.1%)	
Tremelimumab	NCT02527434	Tremelimumab monotherapy	32	93.8%	Fatigue (28.1%)	59.4%	Colitis (9.4%)	NONE
					Colitis (25.1%)		Anemia (9.4%)	
					Pruritis (21.9%)			
					Diarrhea (18.8%)			
					Nausea (18.8)			

Abbreviations: AEs, adverse effects; AST, aspartate aminotransferase; ALT, alanine aminotransferase; GGT, γ-glutamyl transpeptidase; NR, not reached or not reported.

## IrAEs in Treatment With CTLA-4 Inhibitors

In CheckMate 032, the incidences of AEs at any grades in nivolumab monotherapy group (N3), nivolumab 3 mg/kg plus ipilimumab 1 mg/kg group (N3I1), nivolumab 1 mg/kg plus ipilimumab 3 mg/kg group (N1I3) groups were 84.6, 84.6 and 80.4%, respectively, while AEs beyond grade 3 were 31.7, 39.1 and 17.8%. The most common AEs mainly occurred in the skin and gastrointestinal system, including diarrhea (32.6%), pruritus (31.5%), fatigue (26.1%), decreased appetite (16.3%) and maculopapular rash (16.3%). Compared with N3I1 group, N1I3 groups demonstrated higher incidence of high-grade AEs, which may be caused by ipilimumab dose-related toxicity (Sharma et al., 2019). Durvalumab plus tremelimumab combine therapy was designed in DANUBE trial, and the incidence of AEs at any grades and grade ≥3 were 74.7 and 27.9%, both of which were intermediate between durvalumab monotherapy and chemotherapy group. The top five AEs were almost the same as nivolumab plus ipilimumab combine therapy group in CheckMate 032, such as pruritus (22.9%), diarrhea (21.1%), rash (15%), fatigue (14.4%) and decreased appetite (7.6%). Two deaths due to study drug toxicity were reported in durvalumab plus tremelimumab group (septic shock and pneumonitis) (Powles et al., 2020b). Another attempt with durvalumab plus tremelimumab combine therapy in clinical trial (NCT02812420) showed 92.9% patients experienced adverse effects, including amylase increased (28.6%), rash (28.6%), pruritus (25%), alanine aminotransferase increased (21.4%) and aspartate aminotransferase increased (21.4%), and noo deaths related to therapy occurred (Gao et al., 2020). The safety of combination therapy appears to be dose and schedule dependent, indicating researchers should seek the best treatment dose to improve the complementary ability of the two drugs.

Besides two ICIs combine therapy, CTLA-4 was also used in mUC patients with chemotherapy or monotherapy. Regarding to ipilimumab plus chemotherapy (NCT01524991), all enrolled 36 patients occurred adverse AEs, while 80.6% of them experienced AEs at grade 3 or 4. Fatigue, nausea, constipation, anemia and diarrhea are the most common adverse reactions with the incidences exceed 64% (Galsky et al., 2018). NCT02527434 was conducted to explore the efficacy and safety of tremelimumab monotherapy. Final data revealed that 93.8% patients occurred AEs at any degrees, and 59.4% patients experienced AEs beyond grade 3. This monotherapy clinical trial finally provided theoretical support about tremelimumab as a component of combination therapy with an anti–PD1/PD-L1 agent in mUC patients (Sharma et al., 2020).

The success of PD1/PD-L1 blockade has brought an emerging interest in exploring the role of CTLA-4 blockade alone, and in combination with PD1/PD-L1. Until now, more and more onging studies are carried out, aiming to identify early adverse reactions and guide better management protocols. Updated ongoing clinical trials’ information of CTLA4 inhibitors in patients with mUC are shown in Table 4.

**TABLE 4:** Ongoing clinical trials’ information of CTLA4 inhibitors in patients with mUC.

Agent	Clinical trial	Phase	Enrolled patients	Treatment arms	Primary endpoints	Estimated study completion date	Status
Nivolumab	NCT03036098 (CheckMate 901)	Phase III	1,290	Nivolumab plus ipilimumab, nivolumab plus gemcitabine and cisplatin	OS, PFS	August 29, 2024	Recruiting
	NCT03844256 (CRIMI)	Phase I/II	50	Nivolumab, ipilimumab, Mitomycin, Capecitabine, Radiotherapy	Toxicity, DLT, DFS	January 2025	Recruiting
	NCT03520491	Phase II		Nivolumab, ipilimumab	RC-PLND	January 2022	Recruiting
Durvalumab	NCT03472274 (DUTRENEO)	Phase II	99	Durvalumab, tremelimumab, Chemotherapy	Antitmor activity	December 2022	Recruiting
	NCT03682068 (NILE)	Phase III	885	Durvalumab plus cisplatin/carboplatin and gemcitabine, durvalumab plus tremelimumab and cisplatin/carboplatin and gemcitabine	PFS, OS	October 30, 2025	Recruiting
	NCT03549715 (NEMIO)	Phase I/II	120	Durvalumab, tremelimumab, ddMVAC	Toxicity, pCR	September 2025	Recruiting
	NCT03702179	Phase II	32	Durvalumab, tremelimumab, Radiotherapy	PaR	December 2022	Active, not recruiting
Tremelimumab	NCT03871036 (ICRA)	Phase I/II	50	Tremelimumab, tremelimumab plus paclitaxel, tremelimumab plus durvalumab and paclitaxel	ORR	February 1, 2023	Recruiting

Abbreviations: DLT, dose limiting toxicity; DFS, disease free survival; RC-PLND, number of patients who proceed to radical cystectomy and pelvic lymph node dissection; pCR, pathologic complete response; PaR, pathological response rate; ORR, overall response rate; PFS, progression-free survival; OS, overall survival.

## Discussion

Over the years, few emerging treatments for mUC have been reported, and chemotherapy is thought to be the most effective therapeutic method for treating mUC patients. Therefore, the advent of immunotherapy has been a boon to patients with mUC. ICIs have achieved unprecedented success in the first and second-line treatments of mUC due to their potent anti-tumor immune reactions and ability to improve the survival rate of patients. However, the adverse effects and toxicity of ICIs cannot be ignored. In this review, we have summarized the current data on AEs of ICIs used to treat mUC, particularly for irAEs.

The mechanism of irAEs has not been fully elucidated. IrAEs are usually associated with the immune system fighting against specific organs or tissues probably due to activation of immune-related T cells, thereby leading to high levels of cytokine secretion by CD4^+^ helper T cells and tissue infiltration by CD8^+^ T cells (Tarhini, 2013). Under the action of immune checkpoint inhibitors, the immune cross-response of T cells acts on tissues and organs, ultimately resulting in adverse reactions similar to autoimmune syndrome (Weber et al., 2015).

A previous study reported that IrAEs occur in approximately 70% of mUC patients treated with ICIs, with the majority occurring between 3 and 6 months during treatment (Postow et al., 2018). Cutaneous irAEs are the most common and present as mild symptoms. The most common skin symptom is maculopapules, which are confined to a small area of the skin and are usually relieved using topical glucocorticoids. When skin lesions are diagnosed as grade 2 or above, oral glucocorticoids may be added or the dosage or duration of immunotherapy medications may be adjusted, or treatment may be discontinued (Diesendruck and Benhar, 2017). Another common irAEs in ICIs were gastrointestinal symptoms characterized by diarrhea. The main manifestations of endoscopic autoimmune colitis were colonic mucosal congestion, edema, or hemorrhage, which could involve the distal colon and rectum. Patients with severe reactions may have intestinal perforation or obstruction, which requires prompt detection and treatment. Grade 1 to 2 diarrhea is usually tolerated or can be relieved using antidiarrhoeic medication. However, patients with advanced diarrhea should take oral glucocorticoids and should be further examined to rule out infection (Martins et al., 2019). Thyroiditis, hypothyroidism, and adrenal insufficiency are common endocrine system irAEs, which suggests that all patients treated with ICIs should be followed up to monitor thyrotropin (TSH) and periodically review thyroid function (Wright et al., 2021). Corticotropic hormone deficiency can cause symptoms such as fatigue, which should also be monitored (Johnson et al., 2020). Moreover, the incidence of immunotherapy-related liver toxicity is relatively low, mainly manifested as elevated transaminase or bilirubin. A small number of patients presented with fever and lymphocyte infiltration was observed on liver biopsy. Therefore, liver function examination should be performed before treatment to rule out liver function injury caused by tumor progression, viral infection, or other drug factors. Treatment should be discontinued for patients with liver function impairment above grade 2, and patients with grade 3 to 4 should receive intravenous glucocorticoid therapy (Cui et al., 2020). Other rare adverse reactions include pulmonary sarcoidosis, immune-associated pancreatitis, nervous system reactions, ocular reactions, and hyperdynamic inflammation. To date, the rate of discontinuation of treatment due to irAEs is low.

Enhanced management of mUC patients during ICIs treatment will help to better screen and prevent occurrence of irAEs. A rigorous clinical examination, including an assessment of baseline condition of each patient, is recommended before ICIs treatment begins. Any new or worsening of existing symptoms must be carefully monitored and, if necessary, the cause should be explored to rule out the possibility that continued immunotherapy may worsen the condition. In addition, early identification and treatment of irAEs is necessary to limit their duration and severity. Therefore, physical, laboratory, and radiographic examinations performed at baseline (before the initiation of immunotherapy) will be used as a reference for any clinical, biological, or radiographic abnormalities that occur during the course of treatment (Mekki et al., 2018; Martins et al., 2019; Johnson et al., 2020). Patients should also be closely monitored throughout the duration of immunotherapy since irAEs may occur at any time, including at the start of treatment, during treatment, and even after the end of treatment. Furthermore, continued monitoring for 1 year after discontinuation of immunotherapy is recommended (Puzanov et al., 2017; Brahmer et al., 2018b; Postow et al., 2018).

We speculate that doctors and patients will gradually be faced with several irAEs as more ICIs are approved and more indications become available. However, it is challenging to diagnose irAEs because the clinical presentations of irRAEs are highly variable (Mehnert et al., 2017). This often results in delayed diagnosis, thereby missing opportunities for early treatment. Therefore, there is an urgent need for identification of biomarkers that can predict irAEs at an early stage. Previous studies have reported that low neutrophil-to-lymphocyte ratio (NLR) < 3, prognostic nutrition index (PNI) ≥ 45, and platelet-to-lymphocyte ratio (PLR) < 180 are associated with a higher incidence of irAEs (Eun et al., 2019; Pavan et al., 2019; Peng et al., 2020). A recent multivariable analysis showed that PLR was more indicative than NLR and PNI. Eosinophils are also being studied because of their role in autoimmune diseases. Several studies have reported that the increase of eosinophils is associated with the incidence and severity of irAEs, which suggests that eosinophils may be an effective biomarker for the diagnosis of irAEs (Jaber et al., 2006; Diehl et al., 2017; Nakamura et al., 2019). B cells have been shown to play an important role in ICIs treatment, and the severity of B cell decline has been reported to be directly associated with the onset time of irAEs (Das et al., 2018; Cabrita et al., 2020; Helmink et al., 2020; Petitprez et al., 2020). Multiple studies have also reported that interleukin-6 (IL-6) and C-reactive protein (CRP) are two promising biomarkers that are associated with irAEs (Mekki et al., 2018; Cui et al., 2020; Johnson et al., 2020; Wright et al., 2021), but the results have been inconsistent in multivariate analyses. Therefore, more meaningful clinical studies are required to elucidate their role. In addition, IL-1B, IL-2, and GM-CSF have been found to be associated with irAEs, and early decrease in IL-8, MCP-1, and G-CSF during treatment were reported to be connected with thyroid irAEs (Kurimoto et al., 2020). Autoantibodies have also been implicated in the development of IRAEs. For example, thyroid peroxidase and thyroglobulin antibodies are associated with thyroid dysfunction after PD-1 treatment (Kimbara et al., 2018; Kurimoto et al., 2020). The level of anti-BP-180 IgG is suspected to be associated with skin irAEs, and anti-CD74 antibodies are expected to be associated with pneumonitis (Tahir et al., 2019; Hasan Ali et al., 2020). Collectively, the above-mentioned studies have reported the potential biomarkers of irAEs. However, their indicative utility needs to be further evaluated due to the small study population, inconsistent clinical measurements, and the diversity of solid tumors. Moreover, the biomarkers should be specifically analyzed in mUC patients due to the immune-specificity of urothelial carcinoma, which differs from other tumors.

## Conclusion and Future Directions

In summary, immunotherapy has been widely applied in mUC treatment. In clinical application, we should pay equal attention to the effectiveness and safety of ICIs. This review has summarized the safety data from all existing clinical trials of ICIs in the mUC treatment field, with particular reference to irAEs. ICIs have demonstrated good safety in mUC treatment, with manageable irAEs. However, doctors should endeavor to acquire more immunological knowledge and make immediate diagnosis based on the manifestations of irAEs. In future studies, baseline values of patients’ indicators should be actively recorded before the administration of ICIs, and reliable biomarkers regarding irAEs should be identified. We hope to improve the efficacy of ICIs, reduce the adverse effects, and allow patients to obtain safe and durable anti-tumor effects through early multidisciplinary intervention and individualized treatment.
